# Effect of pulsed transcranial ultrasound stimulation at different number of tone-burst on cortico-muscular coupling

**DOI:** 10.1186/s12868-018-0462-8

**Published:** 2018-10-03

**Authors:** Ping Xie, Sa Zhou, Xingran Wang, Yibo Wang, Yi Yuan

**Affiliations:** Institute of Electric Engineering, Yanshan University, Qinhuangdao, 066004 Hebei China

**Keywords:** Pulsed transcranial ultrasound stimulation, Cortico-muscular coupling, Number of tone bursts, Mutual information, Transfer entropy

## Abstract

**Background:**

Pulsed transcranial ultrasound stimulation (pTUS) can modulate the neuronal activity of motor cortex and elicit muscle contractions. Cortico-muscular coupling (CMC) can serve as a tool to identify interaction between the oscillatory activity of the motor cortex and effector muscle. This research aims to explore the neuromodulatory effect of low-intensity, pTUS with different number of tone burst to neural circuit of motor-control system by analyzing the coupling relationship between motor cortex and tail muscle in mouse. The motor cortex of mice was stimulated by pulsed transcranial ultrasound with different number of tone bursts (NTB = 100 150 200 250 300). The local field potentials (LFPs) in tail motor cortex and electromyography (EMG) in tail muscles were recorded simultaneously during pTUS. The change of integral coupling strength between cortex and muscle was evaluated by mutual information (MI). The directional information interaction between them were analyzed by transfer entropy (TE).

**Results:**

Almost all of the MI and TE values were significantly increased by pTUS. The results of MI showed that the CMC was significantly enhanced with the increase of NTB. The TE results showed the coupling strength of CMC in descending direction (from LFPs to EMG) was significantly higher than that in ascending direction (from EMG to LFPs) after stimulation. Furthermore, compared to NTB = 100, the CMC in ascending direction were significantly enhanced when NTB = 250, 300, and CMC in descending direction were significantly enhanced when NTB = 200, 250, 300.

**Conclusion:**

These results confirm that the CMC between motor cortex and the tail muscles in mouse could be altered by pTUS. And by increasing the NTB (i.e. sonication duration), the coupling strength within the cortico-muscular circuit could be increased, which might further influence the motor function of mice. It demonstrates that, using MI and TE method, the CMC could be used for quantitatively evaluating the effect of pTUS with different NTBs, which might provide a new insight into the effect of pTUS neuromodulation in motor cortex.

## Background

Neuromodulation techniques have gained attention recent years for both neuroscientific research and neural engineering applications [1, 2]. Pulsed transcranial ultrasound stimulation (pTUS) [3, 4] is a promising technique for neuromodulation which has non-invasiveness, high spatial resolution (< 2 mm), and deep penetration [5–7]. As a mechanical pressure wave, pulsed ultrasound can be transmitted through the skull and facilitate or inhibit neural activities [8, 9]. By observing the cerebral blood flow [10], LFPs or EEG signals from brain [11, 12] or electromyography (EMG) signals from the muscle [13–15], etc., the effect of pTUS have been widely investigated. For instance, Legon W et al. modulated the activity of primary somatosensory cortex and spectral content of sensory-evoked brain oscillations in humans [16]. Li [10] and Guo [17] used low-intensity pTUS to modulate the brain of stroke rats and found pTUS is neuroprotective for ischemic brain injury. Previously, we [11] found that the focused ultrasound stimulation could modulate the phase-amplitude coupling between neuronal oscillations in the rat hippocampus. Moreover, pTUS can stimulate the motor cortex to induce muscle contraction and EMG signals [13]. These rapidly increasing body of findings provide ample evidence that ultrasound stimulation can flexibly modulate the cortical oscillatory dynamics and induce evident motor response.

As a well-established neurophysiological measure, cortico-muscular coupling (CMC) can be used to understand the communication between the oscillation of the cortical and spinal cord activities [18–20]. It is generally believed that the effective movement control depends on the synchronization of oscillatory activity between the motor cortex and effector muscle [21, 22]. By analyzing the coupling between the local field potentials (LFPs) (or magnetoencephalogram (MEG), electroencephalogram (EEG)) of the motor cortex and the electromyogram (EMG) of muscles, previous studies shown that CMC is related to the motor performance [23] and could identify the impaired neural pathway in patients [24]. As pTUS could elicit evident muscle contraction [13] and modulate neural oscillatory [11], we speculate that pTUS-induced change of information flow between motor cortex and effector muscle is subsistent, which could be evaluated by CMC. Previous studies about the effect of pTUS mainly focus on the change of neural activities in the brain [25] or the motor response in muscle [26], however, the coupling between the cortical and spinal cord activities during pTUS is still unknown. Therefore, it is important to evaluate the influence of pTUS with different parameters on neuromodulation from a cortical-muscular coupling view.

As the neural network of cortico-muscular system has nonlinear features of its parts and interactions between them [27], MI [28] and TE [29], which are model-free and sensitive to nonlinear interaction [30], are capable of quantitatively describing the cortico-muscular coupling by measuring the statistical dependencies between two variables [31–33]. In addition, the coupling between cortical and the targeted muscle is bidirectional which includes both the motor command from the cortex and feedback information from the contracting muscle [34, 35]. Because MI is symmetric, it could be used to quantify the amount coupled information of cortico-muscular [33] without the directional information between them [36]. TE which complements the non-directional defect of MI [37], can be used to evaluate the directional interaction of CMC [32].

In the present study, we introduce a novel way to assess the effect of pTUS with different NTBs by applying the cortico-muscular coupling between motor cortex and the tail muscles in mice, thus allowing for quantification of ultrasound effect on motor command circuit. First, since low-intensity pTUS is capable of neuromodulation without thermal effects or tissue damage [8, 38], the low-intensity transcranial ultrasound (1.1 W/cm^2^) was applied to stimulate the motor cortex in mice at different number of tone bursts (NTB = 100, 150, 200, 250, 300). Then, the LFPs in tail motor cortex and EMG in tail muscles were recorded simultaneously during pTUS. Finally, based on the recorded LFPs and EMG signals, the integral coupling strength between cortex and muscle induced by pTUS was evaluated by mutual information (MI), and the change of directional information interaction between them was analyzed using and transfer entropy (TE).

## Methods

### Data recording

#### Experimental system and parameter settings

**Fig. 1:**
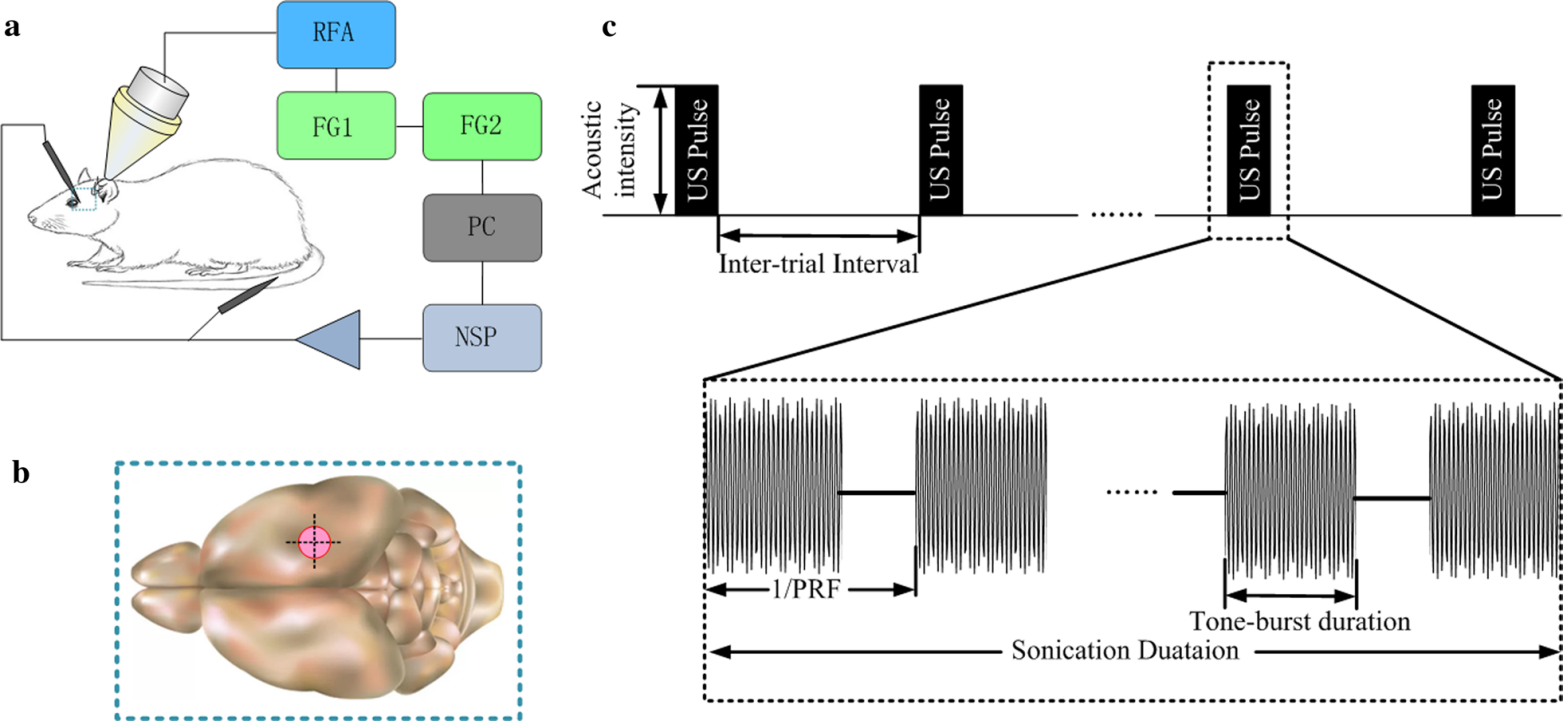
The experimental system (**a**), sonication position (**b**) and parameters used for generating pTUS signal (**c**)

The experimental system is shown in Fig. 1a, consisting of six main components: (1) two function generators (AFG3022C, Tektronix, USA), (2) a linear radio frequency power amplifier (RFA) (240L, ENI Inc., USA), (3) an unfocused ultrasound transducer (V301-SU, Olympus, Japan) with center frequency of 500 kHz and diameter of 31 mm driven by RFA, and (4) an custom conical plastic collimator (Length 50 mm, diameter 2 and 31.2 mm) filled with degassed ultrasound gel and delivering the pTUS to the cortex, (5) single-channel microelectrodes (WE50030.1B10, MicroProbe, USA) recording the LFPs and fine wire electrode recording EMG signals,(6) a dual-channel front-end amplifier (63386, A-M SYSTEMS INC., USA) that amplifying the LFPs and EMG signals, and a 16-channel neural signal processor (NSP) (Cerebus Data Acquisition System, Blackrock Microsystems, USA) converting the signals into digital signals, (7) a computer for data storage and displaying the recorded data simultaneously.

Ultrasonic parameters are illustrated in Fig. 1c, i.e., acoustic intensity (AI), the number of acoustic cycles per pulse (NC), pulse repetition frequency (PRF), the number of tone bursts (NTB), the inter trial interval (ITI) and the sonication duration. In this paper, the parameter setting is AI = 1.10 W/cm^2^, NC = 250, PRF = 1 kHz, ITI = 3.6 s. The excitability or inhibition of pTUS on the neural oscillatory activity are related to the ultrasound beam and parameters of ultrasound [17], especially the pulse repetition frequency. Based on our experiments and other literatures [10, 17, 39], we used PRF = 1kHz to facilitate the motor cortical activity and evoke EMG signals in tail muscle. To explore the effect of pTUS to cortico-muscular coupling, the sonication duration was changed with different NTB (100, 150, 200, 250, 300). High-intensity and long duration ultrasound stimulation can produce thermal effects and damage brain tissue [40]. Therefore, it is safe to use low-intensity pTUS with NTB = 100, 150, 200, 250, 300 in the present study [39]. The pTUS signals were digitized at a sample rate of 30 kHz.

#### Animal surgery and anesthesia

Nine BALB/c mice (male, body weights ~ 20 g, Beijing Vital River Laboratory Animal Technology Co., Ltd. China) were used in this study. After anesthetized with sodium pentobarbital (1%, 5 mg/100 g, IP), mice were constrained on the stereotaxic apparatus (68002, 68030, RWD Co., China). Then, the fur covering the scalp was shaved and the skin was cleaned with physiological saline solution. The scalp of the mice was incised along the midline of the skull, and the exposed tissues and periosteum were cleaned carefully to expose the skull. Finally, the sonication site as illustrated in Fig. 1b, was determined by an atlas and a cranial window of ~ 0.5 × 0.5 cm was drilled to expose the brain tissue in the tail motor cortex. At the end of the experiment, mice were sacrificed with an overdose anesthetic (sodium pentobarbital, 1%, 15 mg/100 g, IP). All the experiment steps were approved with the Animal Ethics and Administrative Council of Yanshan University, Hebei Province, China.

#### Data acquisition

After the surgery procedure, a tungsten microelectrode was inserted into the tail motor cortex to acquire the LFPs signal, a fine-wire was inserted into tail muscle to acquire the EMG signal. When the anesthesia effect in mice was over, the LFPs and EMG signals were synchronously recorded at 2 kHz using the same device. The angle between the pTUS and microelectrode was ~ 60°. The acoustic collimator connected with the planar ultrasound transducer was aimed at the mice tail motor cortex. The ultrasonic wave passed through the acoustic collimator to stimulate the brain tissue for non-invasive neuromodulation.

### Data processing and analysis

#### Data preprocessing

**Fig. 2:**
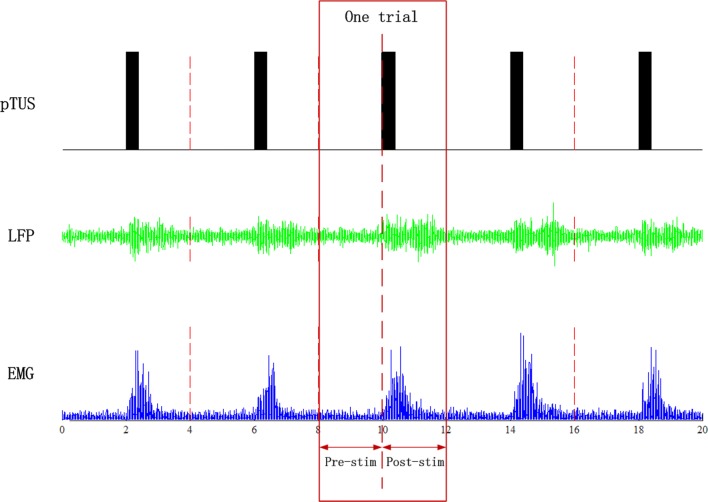
pTUS and the LFPs and EMG signals after preprocessed. The ‘Pre-stim’ represents the time series recorded before stimulation. Reversely, ‘Post-stim’ represents the time series recorded after stimulation

To reject the artifacts in raw LFPs and EMG recordings, a notch filter was used to remove the power signal of 50 Hz and an adaptive high-pass filter was used to remove baseline drift. The LFPs and EMG was band-passed to 0.5–200 Hz and 10–200 Hz, respectively. Then, the EMG was rectified. Finally, the LFPs and EMG before and after stimulation were cut in trials according to the pulse of TUS. After preprocessing, the LFPs, EMG and pTUS were shown in Fig. 2, were used subsequent analysis.

#### Cortico-muscular coupling analysis by mutual information

In this paper, the amount coupled information of cortico-muscular under pTUS was quantitively described by mutual information [28]. The LFPs and EMG were denoted as *x*_*t*_ and *y*_*t*_, respectively. The entropy of LFPs could be computed as following:

1 $$ H(LFP) = - \int\limits_{x} {p(x)\log (p(x))dx} $$

where *p*(*x*) is the probability density function of LFPs. The entropy of EMG can be calculated as the same way.

The joint entropy of LFPs and EMG is:

2 $$ H(LFP,EMG) = - \int\limits_{x} {\int\limits_{y} {p(x,y)\log (p(x,y))dxdy} } $$

where *p*(*x*, *y*) is the joint probability density function of LFPs and EMG.

The mutual information between LFPs and EMG is:

3 $$ MI(LFP,EMG) = H(LFP) + H(EMG) - H(LFP,EMG) = \int\limits_{x} {\int\limits_{y} {p(x,y)\log \frac{p(x,y)}{p(x)p(y)}} } $$

#### Cortico-muscular coupling analysis by transfer entropy

The directional interaction of CMC under pTUS was represented by transfer entropy [29]. Two time series *x*_*t*_ and *y*_*t*_ were approximated by Markov process, the transfer entropy from LFPs to EMG under pTUS can be written as follows:

4 $$ TE_{LFP \to EMG} = H\left( {y_{t + 1} |y_{t}^{n} } \right) - H\left( {y_{t + 1} |x_{t}^{n}, y_{t}^{n} } \right) = \sum\limits_{{y_{t + 1}, y_{t}^{n}, x_{t}^{m} }} {p\left( {y_{t + 1} ,y_{t}^{n} ,x_{t}^{m} } \right)} \log \left( {\frac{{p\left( {y_{t + 1} |y_{t}^{n}, x_{t}^{m} } \right)}}{{p\left( {y_{t + 1} |y_{t}^{n} } \right)}}} \right) $$

where $x_{t}^{m} = (x_{t} , \ldots ,x_{t - m + 1} )$ and $y_{t}^{n} = (y, \ldots ,y_{t - n + 1} )$, *m* and *n* are the orders of Markov process. $H(y_{t + 1} |y_{t}^{n} )$ is the conditional entropy of EMG depending on the past values.

The two processes LFPs and EMG are reconstructed to a higher and same dimensional space. Thus, the formula of transfer entropy for two time series can be written as follows [41]:

5 $$ TE_{LEP \to EMG} = \sum\limits_{{y_{t + u} ,y_{t}^{d} ,x_{t}^{d} }} p{\left( {y_{t + 1} ,y_{t}^{d} ,x_{t}^{d} } \right)\log \left( {\frac{{p\left( {y_{t + u} |y_{t}^{d} ,x_{t}^{d} } \right)}}{{p\left( {y_{t + u} |y_{t}^{n} } \right)}}} \right)} $$

where $x_{t}^{d} = (x_{t} ,x_{t - \tau } ,x_{t - 2\tau } , \ldots ,x_{t - (d - 1)\tau } )$ and $y_{t}^{d} = (y_{t} ,y_{t - \tau } ,y_{t - 2\tau } , \ldots ,y_{t - (d - 1)\tau } )$. The *d*, *τ* and *u* are the embedding dimension, embedding delay and the prediction time, respectively. The transfer entropy from EMG to LFPs is *TE*_*EMG*→*LFP*_ computed by the same process.

In this paper, the values of mutual information and transfer entropy were calculated using TRENTOOL toolbox [42]. Specifically, The embedding delay (*τ*) and embedding dimension (*d*) for state space reconstruction were determined according to Ragwitz criterion [43]. The Kraskove-Stögbauere-Grassberger estimator and the nearest-neighbor search were applied to perform the TE estimation [44]. The number of neighbors *k* was set to 4 as suggested in [45]. The prediction time *u* was optimized in the range of [10, 49] ms according to the influence of pTUS to EMG responses latency [13].

#### Statistical analysis

The differences between the TE/MI values of pre-stimulation and post-stimulation were statistically analyzed based on one-way repeated measures analysis of variance (rANOVA), and the differences between the TE values of the descending direction and ascending direction also performed by one-way rANOVA. The correlations between LFPs/EMG and MI/TE values at different NTB were determined using PEARSONs Correlation coefficient. The correlation was calculated using the MI/TE values and mean values of LFPs/EMG data in each trial. Significance level was set as p < 0.05. All the results of MI and TE were expressed as mean ± S.D. SPSS 19.0 for windows (SPSS Inc., Chicago, IL, USA) was used for all statistical computations.

## Results

### MI result

**Fig. 3:**
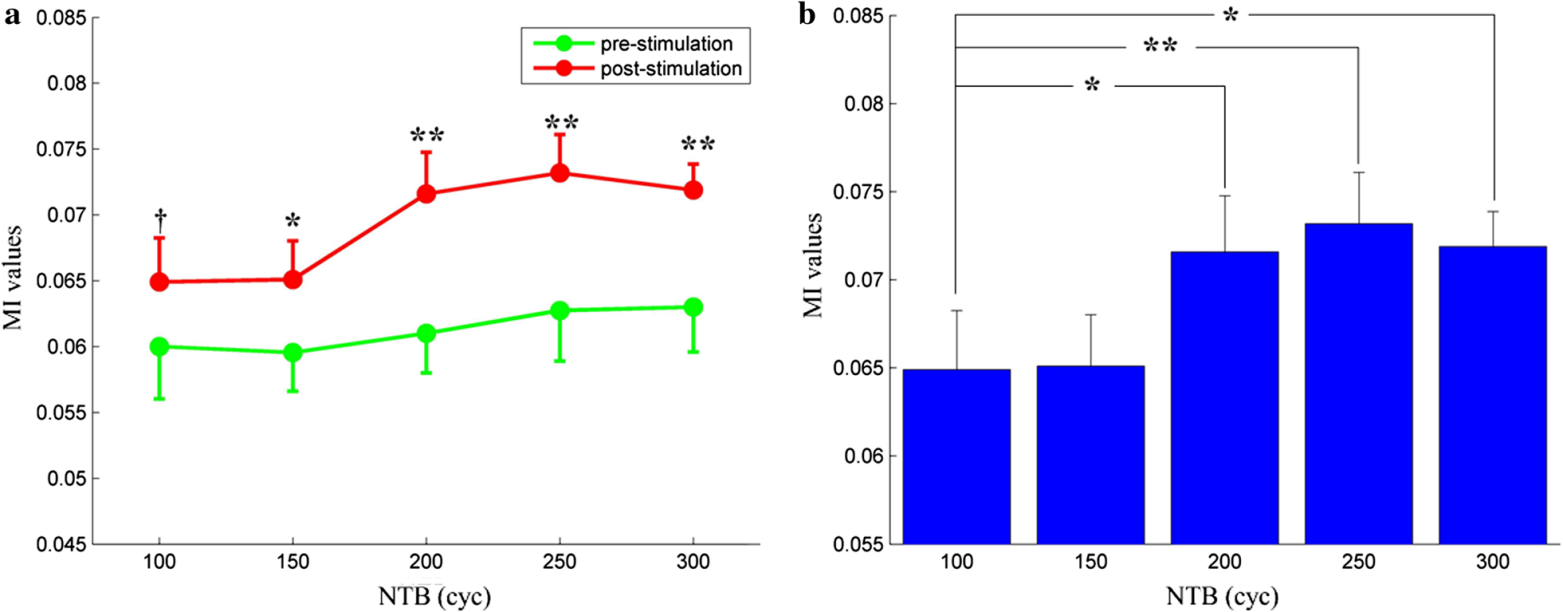
The effects of pTUS to the mutual information between LFPs and EMG. **a** The results of MI before (green line) and after (red line) ultrasound stimulation. **b** The results of MI under pTUS of NTB = 100, 150, 200, 250, 300. *p < 0.05, **p < 0.01, †p = 0.05

To investigate the interaction information between motor cortex and tail muscle, the mean MI values between LFPs and EMG acquired from nine mice were calculated. Figure 3a shows the results of MI between LFPs and EMG before and after stimulation. Before the motor cortex was stimulated by the pTUS, the MI values between LFPs and EMG at different NTB were 0.0600 ± 0.0040,0.0595 ± 0.0029,0.0610 ± 0.0030,0.0627 ± 0.0038,0.0630 ± 0.0034 (mean ± S.D, n = 9). After the motor cortex was stimulated by the pTUS, the MI values were 0.0649 ± 0.0034, 0.0651 ± 0.0030,0.0716 ± 0.0032,0.0732 ± 0.0029,0.0719 ± 0.0020 (mean ± S.D, n = 9). There were highly significant differences (p < 0.01, one-way ANOVA) of MI between before and after stimulation in descending direction at NTB = 200, 250, 300 cyc, while lower significant differences (p < 0.05) of MI between before and after stimulation in ascending direction at NTB = 150 cyc, and no significant difference (p > 0.05) between them when NTB = 100 cyc.

To further explore the influence of pTUS at different NTB on MI values, we performed a significant test with the post-stimulation MI results. As shown in Fig. 3b, when NTB = 200, 250, 300, the MI results were significantly increased (p < 0.05, one-way ANOVA) compared with NTB = 100.

### TE result

**Fig. 4:**
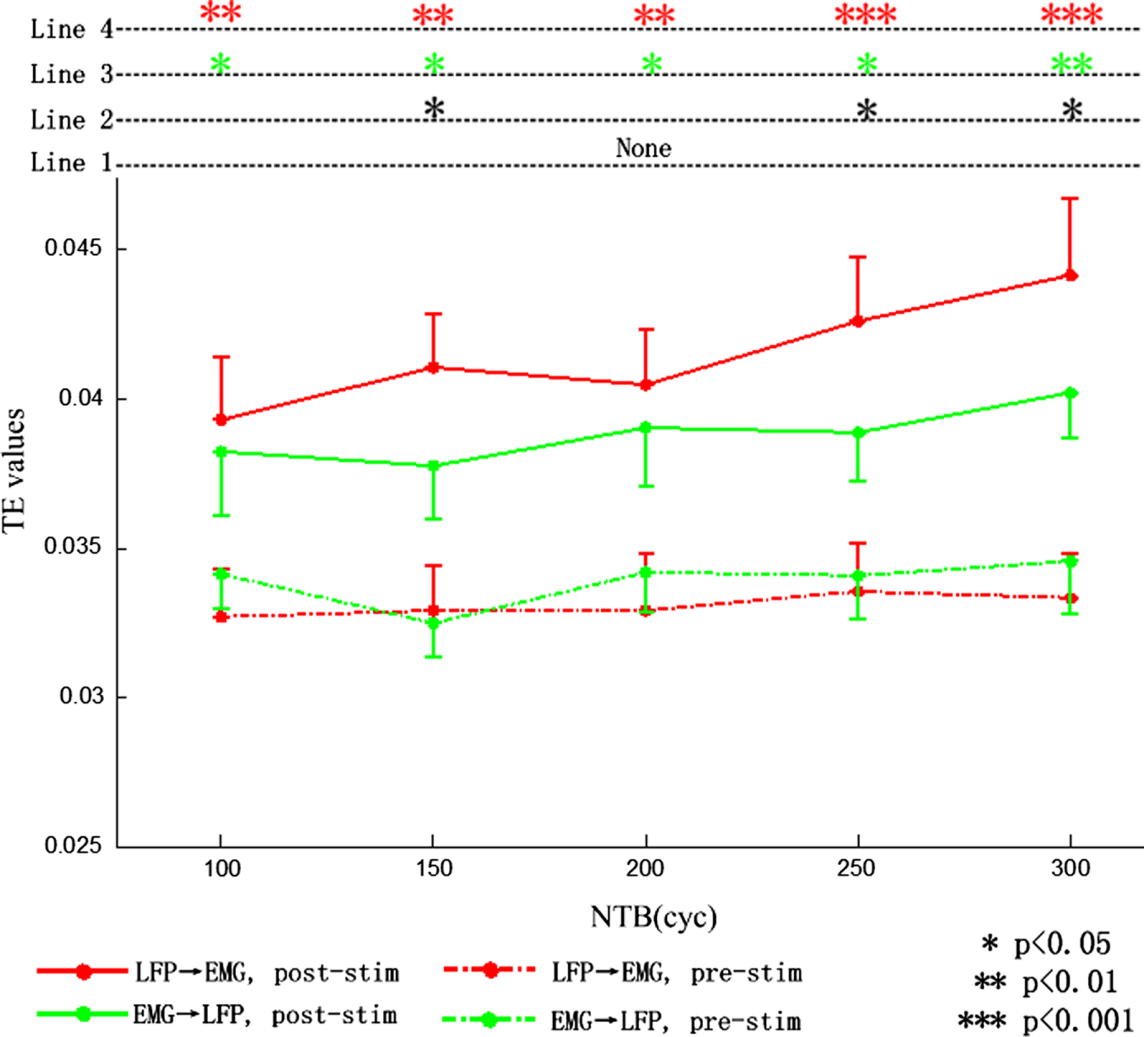
The TE values between LFPs and EMG before and after stimulation. The “Line 1” showed the significant level between descending and ascending direction before ultrasound stimulation, the “Line 2” was after ultrasound stimulation. The Line 3 and Line 4 indicated the significant level between before and after stimulation of ascending direction (from EMG to LFPs) and descending direction (from LFPs to EMG), respectively

To study the changes of directional interaction information between motor cortex and tail muscle that was induced by pTUS with different NTB, we calculated the transfer entropy in both descending (from LFPs to EMG) and ascending (from EMG to LFPs) directions. Figure 4 shows the TE results in descending and ascending direction before and after stimulation. Before the motor cortex was stimulated by the pTUS, the TE values from LFPs to EMG at different NTB were 0.0327 ± 0.0016,0.0329 ± 0.0015,0.0329 ± 0.0019,0.0335 ± 0.0016,0.0333 ± 0.0015 (mean ± S.D, n = 9). And the TE values from EMG to LFPs at different NTB were 0.0341 ± 0.0012,0.0325 ± 0.0011,0.0342 ± 0.0013,0.0340 ± 0.0014,0.0346 ± 0.0018 (mean ± S.D, n = 9).

After the motor cortex was stimulated by the pTUS, the TE values in descending direction were 0.0393 ± 0.0021,0.0410 ± 0.0018,0.0404 ± 0.0019,0.0426 ± 0.0021,0.0441 ± 0.0026 (mean ± S.D, n = 9). The TE values in ascending direction were 0.0382 ± 0.0021,0.0377 ± 0.0018,0.0390 ± 0.0019,0.0388 ± 0.0016,0.0402 ± 0.0015 (mean ± S.D, n = 9). Moreover, the TE values in both two directions were increased after the motor cortex was exposed to pTUS.

The significant analysis (the four lines at the top of Fig. 4) showed highly significant differences (p < 0.01, one-way rANOVA) of TE between before and after stimulation in descending direction (Line 4), while lower significant differences (p < 0.05, one-way rANOVA) of TE between before and after stimulation in ascending direction (Line 3). Additionally, there were three significant differences (NTB = 150, 250, 300 cyc) between the TE in descending and ascending direction after stimulation (Line 2), while no significant difference between them before stimulation (Line 1).

**Fig. 5:**
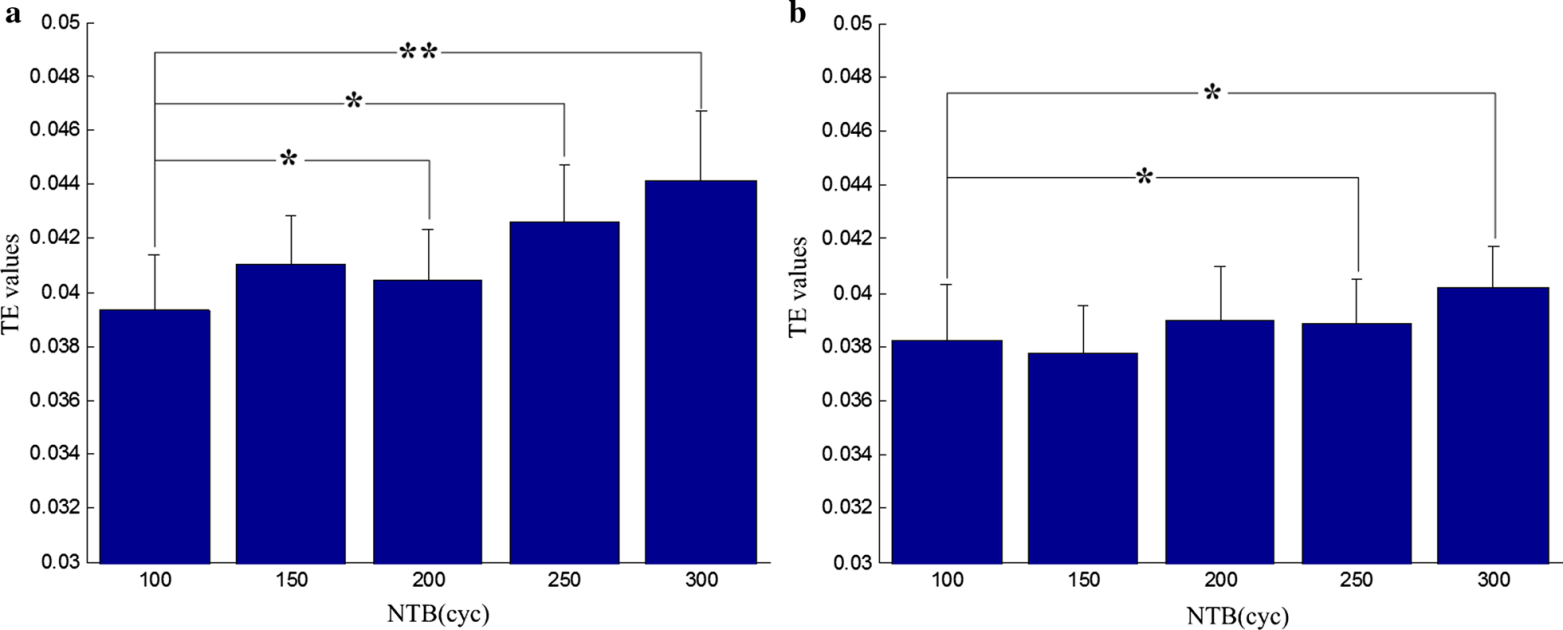
The effects of pTUS with different NTB on the transfer entropy between LFPs and EMG. **a** The results of transfer entropy of descending direction (from LFPs to EMG). **b** The results of transfer entropy of ascending direction (from EMG to LFPs). “*” denotes p < 0.05, “**” denotes p < 0.01

The effect of different parameters of pTUS to the transfer entropy between LFPs and EMG was shown in Fig. 5. In Fig. 5a, when NTB = 200, 250, 300 cyc, the TE values in descending direction were significantly increased (p < 0.05, one-way rANOVA) compared with NTB = 100 cyc, where the most significant increase (p < 0.01) was in NTB = 300 cyc. In Fig. 5b, when NTB = 250, 300 cyc, the TE values in ascending direction were significantly increased (p < 0.05) compared with NTB = 100 cyc.

## Discussion

Ultrasound stimulation has emerged as a potential approach that can address the defects faced by modern neuromodulation technologies [7, 46], which can be applied noninvasively to activate or modulate the activity of targeted brain regions [16, 47, 48]. Recent years, many studies found evidently motor responses in animals by activating the primary motor cortex using the transcranial ultrasound [5, 13, 14, 49, 50], where the induced movement was all measured by EMG signals. However, both motor command from brain and feedback from muscle are involved in effective movement control [22, 23], and there is a coupled relationship between the cortical oscillation and muscle activation [51, 52]. To our knowledge, there are still a lack of evidence that assessed the neuromodulatory effect of pTUS from the neural circuit of motor-control system view. Thus, in this study, we considered applying the cortico-muscular coupling to evaluate the effect of pTUS with different number of tone bursts (NTB). Since CMC has been applied to assess the movement response induced by other neuromodulation techniques, such as transcranial magnetic stimulation (TMS), transcranial alternating current stimulation (tACS) and deep-brain stimulation (DBS) [53–55], we assume that the CMC could serve as a promising tool for the assessment of ultrasound neuromodulation.

Intention of the present study is to elucidate the effect of pTUS with different number of tone bursts (NTB) using CMC. We recorded LFPs and EMG evoked by pTUS in mice’s motor cortex and tail muscle. As shown in Fig. 2, the amplitude of LFPs and EMG signals increased after stimulation. It means that the neural activity of motor cortex and the contralateral muscle could be altered by pTUS, which supports the previous studies of pTUS [5, 13, 49]. Then, we analyzed the coupling relationships between these two kind of signals using mutual information and transfer entropy.

We can see that both the TE and MI values between LFPs and EMG signals could be significantly increased with pTUS. These results indicated that the CMC between motor cortex and tail muscle could be enhanced by pTUS. Although the reason of the enhancement of CMC induced by pTUS is still unclear, the pTUS-induced EMG response [13] and the cortical excitement [56] might be related to this phenomenon, as significant correlations (p < 0.05) between the mean amplitude of the recorded signals (LFPs and EMG) and MI values could be observed when NTB = 100, 200, 250, 300 (Table 1), where the quality of the correlation was expressed by ρ, and the significant level was expressed by p.

**Table 1:** Results of correlation analysis

NTB	Correlation LFPs and MI	Correlation EMG and MI	Correlation LFPs and TE	Correlation LFPs and TE
			(LFPs → EMG)	(EMG → LFPs)
100	ρ^2^ = 0.032	ρ^2^ = 0.014	ρ^2^ = 0.010	ρ^2^ = 0.012
	p = 0.035	p = 0.043	p = 0.0061	p = 0.058
150	ρ^2^ = 0.010	ρ^2^ = 0.001	ρ^2^ = 0.023	ρ^2^ = 0.002
	p = 0.062	p = 0.086	p = 0.0046	p = 0.084
200	ρ^2^ = 0.036	ρ^2^ = 0.032	ρ^2^ = 0.026	ρ^2^ = 0.0001
	p = 0.026	p = 0.036	p = 0.0043	p = 0.096
250	ρ^2^ = 0.036	ρ^2^ = 0.036	ρ^2^ = 0.090	ρ^2^ = 0.003
	p = 0.024	p = 0.034	p = 0.0015	p = 0.081
300	ρ^2^ = 0.040	ρ^2^ = 0.023	ρ^2^ = 0.292	ρ^2^ = 0.026
	p = 0.0031	p = 0.0047	p = 0.003	p = 0.043

The MI results revealed that the CMC in the sensory and motor system could be enhanced by pTUS (Fig. 3a). The TE results suggested that the CMC in descending direction could be significantly higher than that in ascending direction after stimulation (Fig. 4). It suggested that the neural pathways responded for motor command would transmit more information than the feedback pathway due to the effect of pTUS. Correlation analysis (Table 1) showed a highly significant correlation (p < 0.01) between the mean LFPs amplitude and the TE values in descending direction (LFPs → EMG) (NTB = 150, 200, 250, 300), and a poor correlation (p > 0.05) in ascending direction (LFPs → EMG). The results revealed that the transferred information from the brain to muscle might be facilitated by the excitement of neural activity in motor cortex. It suggested that CMC could serve as a more useful tool for evaluating the effect of pTUS in motor cortex, which could not only assess the pTUS-induced motor responses as previous studies did by using LFP and EMG [12–14], but also reveal the information interaction between motor cortex and muscle in motor system. The mechanism of cortical excitement evoked by pTUS is still debated [57, 58]. In general, cavitation of neural membrane is known as the critical factor for eliciting neuromodulatory efficacy, which has been confirmed in cellular-scale and in vivo [50]. Recent findings revealed an indirect auditory mechanism for ultrasound-induced cortical activity and movement [57, 58]. And we speculate that the no-task experimental condition in mouse, which was different from human [35], might also result in the lower CMC in ascending direction compared with another direction. Overall, the results in this study revealed that MI could be applied to quantitatively estimate the integral CMC between motor cortex and contralateral muscle during pTUS in mouse. And TE could be used to analyze the change of directional interaction information between them. Moreover, the CMC estimated by MI and TE could increase with the increasing of NTB (NTB = 100, 150, 200, 250, 300) (Figs. 3b, 5). As the sonication duration depends a lot on the NTB, this study reveals a positive correlation between CMC and stimulus duration. It also supports that the longer stimulus duration increases the probability of motor response [26, 39].

Furthermore, previous studies showed that the motor response induce by other brain stimulation techniques such as TMS, tACS, DBS could be assessed by CMC in human [53–55], especially in patients with motor dysfunction diseases. For example, the CMC of Parkinson’s disease (PD) could be modulated by DBS [59]. And pTUS had shown cerebral protection effect for stroke [10]. In this study, the CMC in descending direction is significantly increased and higher than the ascending direction after ultrasound stimulation (Fig. 5a). As it is generally believed that the impairment in neural-pathway of the descending direction is the main cause of stroke [35], applying CMC into the evaluation of pTUS neuromodulation may provide an evidence for understanding the mechanism of pTUS in stroke rehabilitation. Moreover, the MI and TE methods, which quantified CMC, could be used for measuring the effect of ultrasound stimulation and optimizing the ultrasonic duration.

Since this study only explored the influence of pTUS on the CMC in healthy mouse, we plan to extend this work to stroke or PD mice to investigate whether the abnormal CMC in those diseases can be improved by pTUS. Additionally, a previous study suggested that the ultrasound-induced EMG signals in mouse could increase as a function of both the ultrasound intensity and sonication duration [26]. Our study only investigated the effect of sonication duration to CMC, perhaps other parameters such as ultrasound intensity, frequency or number of cycles could also produce modulation effects to CMC. The influence of the ultrasound parameters to CMC could be systematically studied in the next step.

## Conclusion

In this study, the neuromodulatory effect of pulsed transcranial ultrasound was analyzed by the cortico-muscular coupling between motor cortex and tail muscle in mice, which was quantified using the transfer entropy and mutual information algorithms. The results of MI and TE showed that the CMC between motor cortex and tail muscle was significantly increased by pTUS, and the CMC in descending direction could be significantly higher than that of ascending direction after ultrasound stimulation. Furthermore, by increasing the NTB, the CMC between motor cortex and tail muscle could also be significantly enhanced. Since the CMC is a promising tool for movement evaluation, it suggests that pTUS might influence the motor function of mice. This study demonstrates for the first time, using MI and TE method, the CMC can be used for quantitatively evaluating the effect of different sonication durations of pTUS-induced movement, which might provide a new insight into the effect of pTUS neuromodulation in motor cortex.

## Abbreviations

pTUS: pulsed transcranial ultrasound stimulation
EMG: electromyograms
CMC: cortico-muscular coupling
NTB: number of tone bursts
MI: mutual information
TE: transfer entropy
LFPs: local field potentials
MEG: magnetoencephalogram
EEG: electroencephalogram
FG: function generators
RFA: radio frequency power amplifier
NSP: neural signal processor
AI: acoustic intensity
NC: number of acoustic cycles per pulse
PRF: pulse repetition frequency
ITI: inter trial interval
TMS: transcranial magnetic stimulation
tACS: transcranial alternating current stimulation
DBS: deep-brain stimulation
PD: Parkinson’s disease
